# Motor Consciousness during Intention-Based and Stimulus-Based Actions: Modulating Attention Resources through Mindfulness Meditation

**DOI:** 10.3389/fpsyg.2012.00290

**Published:** 2012-09-11

**Authors:** Yvonne Nathalie Delevoye-Turrell, Claudie Bobineau

**Affiliations:** ^1^URECA Laboratory, Université de Lille 3, Université Lille Nord de FranceVilleneuve d’Ascq, France

**Keywords:** grip force, sensation, consciousness, attention, dual task, mindfulness, motor planning, intention

## Abstract

Mindfulness-Based Stress Reduction meditation (MBSR) may offer optimal performance through heightened attention for increased body consciousness. To test this hypothesis, MBSR effects were assessed on the simple task of lifting an object. A dual task paradigm was included to assess the opposite effect of a limited amount of attention on motor consciousness. In a stimulus-based condition, the subjects’ task was to lift an object that was hefted with weights. In an intentional-based condition, subjects were required to lift a light object while imagining that the object was virtually heavier and thus, adjust their grip voluntarily. The degree of motor consciousness was evaluated by calculating correlation factors for each participant between the grip force level used during the lift trial (“lift the object”) and that used during its associated reproduce trial (“without lifting, indicate the force you think you used in the previous trial”). Under dual task condition, motor consciousness decreased for intention- and stimulus-based actions, revealing the importance of top-down attention for building the motor representation that guides action planning. For MBSR-experts, heightened attention provided stronger levels of motor consciousness; this was true for both intention and stimulus-based actions. For controls, heightened attention decreased the capacity to reproduce force levels, suggesting that voluntary top-down attention interfered with the automatic bottom-up emergence of body sensations. Our results provide strong arguments for involvement of two types of attention for the emergence of motor consciousness. Bottom-up attention would serve as an amplifier of motor-sensory afferences; top-down attention would help transfer the motor-sensory content from a preconscious to a conscious state of processing. MBSR would be a specific state for which both types of attention are optimally combined to provide experts with total experiences of their body in movement.

## Introduction

Mental skills are crucial in ensuring great performances at world championships and Olympic Games (Werthner, 2002). The often-referenced study by Orlick and Partington (1988), an extensive study of 235 Canadian athletes, revealed that mental readiness was a significant factor in determining which athletes were able to perform their best under the pressures and stresses of the Olympics. The ability to focus attention and control performance imagery was later found to be the key factors in successful performances (Werthner, 2002). What a person directs his or her attention to while preparing to execute a skill determines in fact how fluid the motion, how consistent the movement and in general, how accurate the outcome is (for a review, see Wulf et al., 2010b).

In the past 20 years, mindfulness-based stress reduction (MBSR) meditation has been adopted by many athletes especially those evolving in individual sports for which self-awareness of body performance is a key to success (skiing, gymnastics, swimming). Acquired through long periods of months, some athletes are convinced of its usefulness for filtering in only those positive thoughts needed for the task at hand, thus augmenting concentration capacities. Others are more sensitive to the role of mindfulness to heighten the *sensory experiences* of their body during performance execution. The aim of the present study was to develop a simple lab-based methodology to assess objectively the effects of mindfulness meditation through the manipulation of the nature of the task (automatic vs. controlled) and of the quantity of available attention (simple vs. dual task) during the planning of a simple grip to lift action. More specifically, we wanted to gain a better understanding of the role played by meditation for the allocation of attention resources in relation to the emergence of the consciousness of body in action.

### Meditation for heightened motor attention

Even if grip force (GF) is a motor parameter that is scaled automatically, it appears that during some fine manipulative tasks (e.g., threading a needle), we can make a conscious effort to orient more attention to modulate muscle contraction output (Delevoye-Turrell and Wing, 2004). As such, it is possible to increase our levels of body consciousness of even the smallest body part (*while reading this sentence, orient in the present moment your attention to your left big toe*…). With practice, it has been shown that body consciousness can become so vivid and intense that certain experts report having experienced what is called the flow state of consciousness – an optimal performance state that is most searched for by athletes (Bianco et al., 1999) and by music maestros (Glise, 2011). During flow state, the modified state of attention can increase body consciousness to such an extent that performers experience loss of temporal awareness, a calm loss of emotional and physical tension as well as an increase sensitivity to sound, light, and tactile stimuli (Williams and Krane, 1993). Because of these observations, it has been suggested that MBSR meditation techniques may help individuals gain control on those brain mechanism leading to the emergence of the flow state of body consciousness.

In current research contexts, MBSR meditation is typically defined as non-judgmental attention to experiences in the present moment (Shapiro et al., 2008). Bishop et al. (2004) have suggested a two-component model of mindfulness where the first component is the regulation of attention in order to maintain it on the immediate experience, and the second component involves approaching one’s experiences with an orientation of curiosity, openness, and acceptance. As such, the practice of mindfulness meditation encompasses *paying attention on purpose, in the present moment, on the experience of thoughts, emotions, and body sensations simply observing them as they arise and pass away* (Kabat-Zinn, 1994). Attentional training and improvement are in fact core elements in traditional meditation practices, and meditation types are often defined according to their attentional characteristics (Andresen, 2000; Lutz et al., 2008).

Nevertheless, it is also known that paying “too much attention” to our movements can disrupt performance, especially if the skill is well practiced and performed following an automatic *routine* (Flegal and Anderson, 2008). For example, being conscious of our feet when quickly walking down a flight of stairs can make one trip and fall. There is hardly an athlete, a musician, or a public speaker who could not give an example of “chocking,” especially when he or she was trying hard to concentrate and do well (Beilock et al., 2002). It is thus possible that heightened attention to body movements through MBSR techniques may be good for certain types of movements only.

### Two modes of action planning for adapted interaction with the world

It is the case that there are two principal ways in which one can interact with the environment. One may carry out movements to manipulate the environment in order to produce desired environmental effects. In such an intentional state, one may grasp a plastic cup to squash it intentionally before placing it in the bin. In such *intention-guided* case, the force level applied through the fingertips is increased voluntarily to reach the desired state of *a flattened cup*. On another hand, actions may be carried out to accommodate environmental demands: grasp the cup to move it and make place for a hot dish. In this *stimulus-driven* case, the force level applied through the fingertips is increased automatically, without further thought. There is now convincing scientific evidence that intention-guided actions, on the one hand, and stimulus-driven actions, on the other hand, are controlled by different neural-psychological pathways (Herwig et al., 2007; Tubau et al., 2007; Casal et al., 2009).

The activity of these pathways has been shown to rely on different kinds of memory traces (Elsner and Hommel, 2001). The stimulus-based control mode represents a case of a “prepared reflex” (Hommel, 2000), for which the cognitive system is prepared to respond to particular, typically highly response compatible stimuli in a more or less automatic fashion. Accordingly, not much of the sequence is actually learned and little attention resources are thought to be required. In contrast, actions following an internal desire will produce a selected series of actions, and it has been suggested that the intention-based control mode relies on the construction of an action plan (Luria, 1962; Maasen et al., 2003), which consists in the planning of ordered sequences of representations of action effects (Hommel, 1996). Intention-based control implies that plan-related representations (i.e., action-triggering signals) are internally generated (Zelazo et al., 1999) and as such, require attention for optimal preparation and fluent execution.

### Contrasting levels of motor attention in function of action mode

To gain a direct insight on exactly what aspects of movement planning or executing required attention resources, we used a dual task paradigm to probe the levels of attention needed for gripping actions (Delevoye-Turrell et al., 2006). In an implicit scaling condition (stimulus-driven), subjects were required to reach for an object and move it across the table. Because there was an objective to the task, the subjects’ focus of attention was oriented toward the final goal of the task and thus, GF was automatically scaled to the object’s weight. In the explicit scaling condition (intention-based), the subjects’ task was to reach for the object and grip it harder, i.e., increase explicitly the level of GF applied to the surfaces of the object. An auditory probe could occur before or during action execution, and subjects were instructed to react as fast as possible to the probe (with their foot) without interrupting the hand-task. This dual task paradigm provided the means to evaluate the amount of attention used for motor *planning* (with a probe that occurred between the start of the trial and movement onset) and for motor *execution* (with a probe that occurred during the movement) as a proportion of reaction time augmentation (Kahneman, 1973). Results showed that there was a significant increase in reaction times for all types of actions under dual task compared to single task conditions, suggesting that all grip actions required a minimal amount of attention both for planning and execution. Nevertheless, less attention was overall required for the planning of stimulus-driven actions compared to that used for intention-based actions.

Following the idea that more attention should lead to increased levels of motor consciousness (i.e., the explicit knowledge of physical responses), we predicted in the present study that intention-based actions would be associated to a higher level of motor consciousness compared to stimulus-driven actions. Under the effects of MBSR meditation, motor consciousness should be maximal as subjects focus more attention resources during action planning and execution, with however lower reproducing capacities in the stimulus-based mode of action planning. Finally, because attention is required for even the simplest gripping task, the decrease in attention availability (through the use of a dual task paradigm) should impair the levels reached of motor consciousness by all our participants in both modes of action planning.

## Materials and Methods

### Participants

Participants were recruited in the different departments of the University of Lille3 (psychology; musicology; arts) and at the symphony orchestra of Lille (*Orchestre Nationale de Lille*). They were divided in two groups in function of their knowledge and expertise in mindfulness MBSR meditation technique. The group of MBSR-experts were 10 right-handed professional or amateur musicians who practiced daily mindfulness meditation (six males; mean age 40.8 years, SD 11.2, range 23.6–56.2; years of education 14.9, SD 2.6). The mean level of mindfulness meditation experience was high (mean period of 12.2 years, SD 5.2, range 3.0–23.5). Twenty right-handed professional or amateur musicians (eight males; mean age 36.0 years, SD 16.1, range 19.3–62.4; years of education 13.9, SD 3.0) also took part in this study as controls; none had any experience in mindfulness meditation. There were no statistical differences between Groups for Age [*t*(1, 28) = 0.841; *p* = 0.407], years of education [*t*(1, 28) = 0.898; *p* = 0.379], and years of musical practice [*t*(1, 28) = 0.871; *p* = 0.436]. All subjects were naïve to the specific purpose of the experiment.

None of the participants had any known psychological or neurological deficits. They had normal or corrected-to-normal vision. The local ethics committee approved the experimental protocol and all participants provided written informed consent after the procedure had been fully explained. Participants were tested individually, in an isolated room and participated in a single experimental session lasting approximately 45-min.

### Apparatus

**Figure 1:**
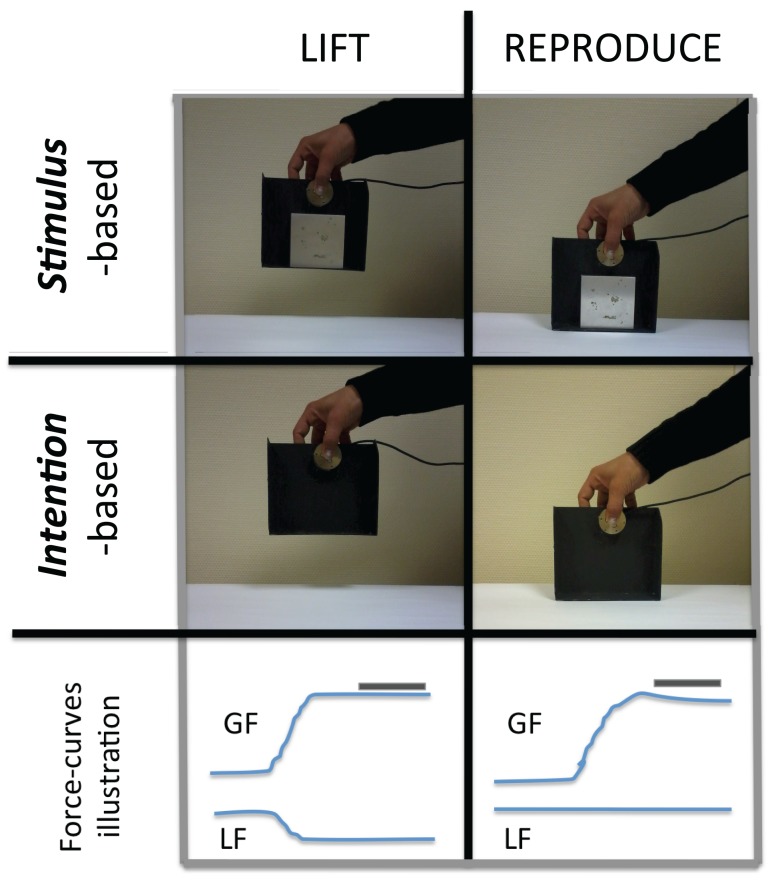
**Pictures of a subject during the LIFT and REPRODUCE-trials for the stimulus-based actions during which the object is hefted with weights (*TOP*), and the intention-based actions during which subjects are required to image that the object is heavier than it is (*MIDDLE*)**. In this later case, subjects are thus required to voluntarily increase the grip force to an imaginary level of force sufficient to lift a virtual object. Force-curves examples are presented (*BOTTOM*) illustrating, for a 3-s trial, the variation of grip force (GF – force applied through the fingers normal to the object’s surfaces), and the variation of load force (LF – force acting tangentially to the object’s surfaces). Note that during the REPRODUCE trials, LF is null, as the object is not lifted off the tabletop. The gray bar illustrates the GF section that was used to calculate the correlation factors that are presented in Figure 2.

Subjects were seated facing a table on which was placed an object. With their dominant right hand, subjects used a precision grip with thumb on one side opposed by three fingers to lift and hold the object (weight: 65 g) that had metal-surfaced plates (see Figure 1). A circular load cell (*TIA* Mini 40 Force/Torques transducer; weight: 50 g; diameter: 40 mm; width: 15 mm) was mounted within this object to measure the GF (in Newton) produced by the digits normal to the vertical grasp surfaces. This six-axis load cell also provided the means to measure load force fluctuations (LF in Newton) that acted tangential to the object’s surfaces, induced by gravity. The load cell was connected to a laptop computer and the data collection was run through custom-made *Labview* software at a sampling frequency of 1000 Hz.

### Experimental procedure

On each trial, participants were required to lift the object and hold it immobile, in mid air, for approximately 3 s (Figure 1 – left). After this LIFT trial, they were instructed to reproduce the GF level they thought they had used on the previous trial to hold the object in mid air (Figure 1 – right). For this REPRODUCE trial, participants were required to maintain the object on the table, thus applying only forces normal to the object’s surfaces. After each pair of trials, the experimenter checked the LF curves to verify that the subjects had followed instructions, i.e., had not lifted the object, even slightly, during the REPRODUCE trial. When the conditions had not been met (LF varied above 0.5 N – see curves in the bottom panel of Figure 1), the pair of trials was discarded; the trial was represented to the participant at the end of the block. The start of each trial was signaled by an auditory beep. The time interval between the end of the LIFT and the start of the REPRODUCE trials was 1.5 s; the time interval between pairs of trials was 5 s.

To gain an insight in the role played by the subjects’ mode of action planning on motor consciousness, two modes of action planning were proposed. In the *stimulus-based* mode, subjects lifted an object that was hefted with a light, a medium-heavy, or a heavy weight in order to afford objects of 200, 400, and 800 g. Hence, the level of active GF required to lift the object depended directly on the levels of passive force induced to the object by the environment (gravity). In the *intention-based* mode, the subjects’ task was to lift the very light object (115 g was the mass of the empty object) and to imagine that the object was light, medium-heavy, or heavy. Hence, under this condition, the active GF scaling depended solely on the force scaling that subjects voluntarily decided to apply. The order in which the two modes were performed was randomly assigned to the subjects who then maintained the same order for the three attention blocks.

To manipulate the quantity of attention resources allocated for the planning phase of the LIFT trial, subjects performed the task in three different attention block conditions. In the *Neutral* condition, subjects performed the LIFT/REPRODUCE pairs of trials without further constraints. For all participants, this was the first condition experienced. Then, either a Heightened (A++) or a Diminished (AA−−) attention block was performed. Under the *Diminished* condition, subjects performed a dual task situation for which they were required to count backward by 7 starting from a three-digit number (e.g., 231). Subjects were required to count as fluently as possible at a tempo of 1 countdown per second, without making a mistake. This count down procedure was performed on the LIFT trial only. The REPRODUCE trial was performed without further constraints. Subjects performed the countdown in their native language to facilitate the task. In this study, five MBSR-experts and two controls performed the task in English. The other participants performed the countdown in French.

Prior to the *Heightened* condition, subjects were asked to relax and become aware of the environmental noises (people walking in the hallway; water dripping in the pipes; airplane passing) in order to anchor themselves in the present moment. Then, the experimenter encouraged all participants to close their eyes and to use the *dominant hand* as specific target of meditation on which to focus attention. If the mind started to wander, subjects were encouraged to simply direct the mind back toward the object of attention with a sense of “friendliness” without judgment. A 5-min meditation program was proposed in order to provide the necessary time for all subjects to become calmer with a slowing down of breathing rhythm and of discursive thought. Without talking, when subjects felt ready to pursue, they opened the eyes and the experimental block was launched.

Subjects performed five pairs of trials for each mass (light; medium-heavy; heavy), in each action mode (stimulus-based; intention-based) under each attention condition (neutral; diminished; heightened) for a total of 90 trials. Prior to the start of the experimental session, it was emphasized that there would be quite a large number of trials to perform (a minimum of 100 trials), and that subjects should relax their grip as best they could to minimize muscle fatigue during the LIFT trials. Subjects were assigned to one of the two experimental groups in function of their knowledge in mindfulness meditation.

At the end of the session, participants were asked to fill in a brief questionnaire to report on their subjective experience of the different attention conditions. In addition, they were asked to score between 1 and 10 the accuracy of reproduction of the force level they thought to have reached, distinguishing between the action modes as well as the attention conditions. Finally, subjects were briefed about the overall aim of the experiment and were thanked for their participation.

### Data analysis

To describe general performance, mean GF levels applied 1.5 s after the start of each trial for a 500 ms-duration was calculated. In the LIFT trials, this time period fell during the time interval for which the object was held immobile in mid air; in the REPRODUCE trials, this time period fell within the time interval for which subjects were indicating force levels used in the previous LIFT trial (see gray horizontal bar in Figure 1 – bottom). To analyze the level of motor consciousness reached by each subject, Pearson correlation analyses were then conducted between the GF level measured for each pair of LIFT and REPRODUCE trials. Finally, these correlation measures were submitted to a 3 by 2 repeated measures Analysis of Variance with Attention (Neutral; Diminished; Heightened) and Mode (Stimulus-based vs. Intention-based) as within subject factors. When required, corrected Scheffé *post hoc* analyses were used. The significance level was set at *p* = 0.05.

## Results

All participants performed the motor tasks under the different attention conditions without apparent difficulty. They also expressed pleasurable experiences in the heightened attention condition, reporting having experienced “calmness” and “control” during the action of lifting.

### Analysis of the GF levels used in the LIFT trials

Statistical analysis on the mean GF used in the LIFT trials revealed an absence of Group differences [*F*(1, 42) = 2.725; *p* = 0.106], with similar force levels for the controls (14.7 SD 0.7 N) and the MBSR-experts (12.2 SD 1.4 N). Furthermore, subjects distinguished well between the three object masses [*F*(2, 42) = 33.584; *p* = 0.001]. For all masses, GF levels were slightly higher in the intention-based mode compared to that observed in the stimulus-based mode but this slight over estimation did not reach significance [*F*(1, 42) = 1.186; *p* = 0.282]. Finally, similar GF levels were used under the different attention conditions [*F*(2, 42) = 1.561; *p* = 0.216]. The detailed values for these results are presented in Table 1.

**Table 1:** **Details of the mean results obtained for the grip force levels (in Newton) used for the different action modes and under the different attention conditions**.

	Neutral (A)		Diminished (A−−)		Heightened (A++)	
	Stimulus	Intention	Stimulus	Intention	Stimulus	Intention
Light	5.9 (2.8)	5.8 (3.4)	8.8 (3.3)	8.5 (3.3)	5.9 (3.2)	4.6 (3.9)
Medium	10.7 (1.3)	13.7 (1.5)	13.7 (1.5)	13.0 (1.5)	11.7 (1.4)	12.2 (1.7)
Heavy	17.8 (1.5)	23.0 (1.8)	20.0 (1.7)	23.5 (1.7)	20.4 (1.6)	22.3 (2.0)

*There were no significant differences between the groups. Thus, results for the 30 participants are presented here averaged together*.

### Effects of attention and planning-mode on motor consciousness

**Figure 2:**
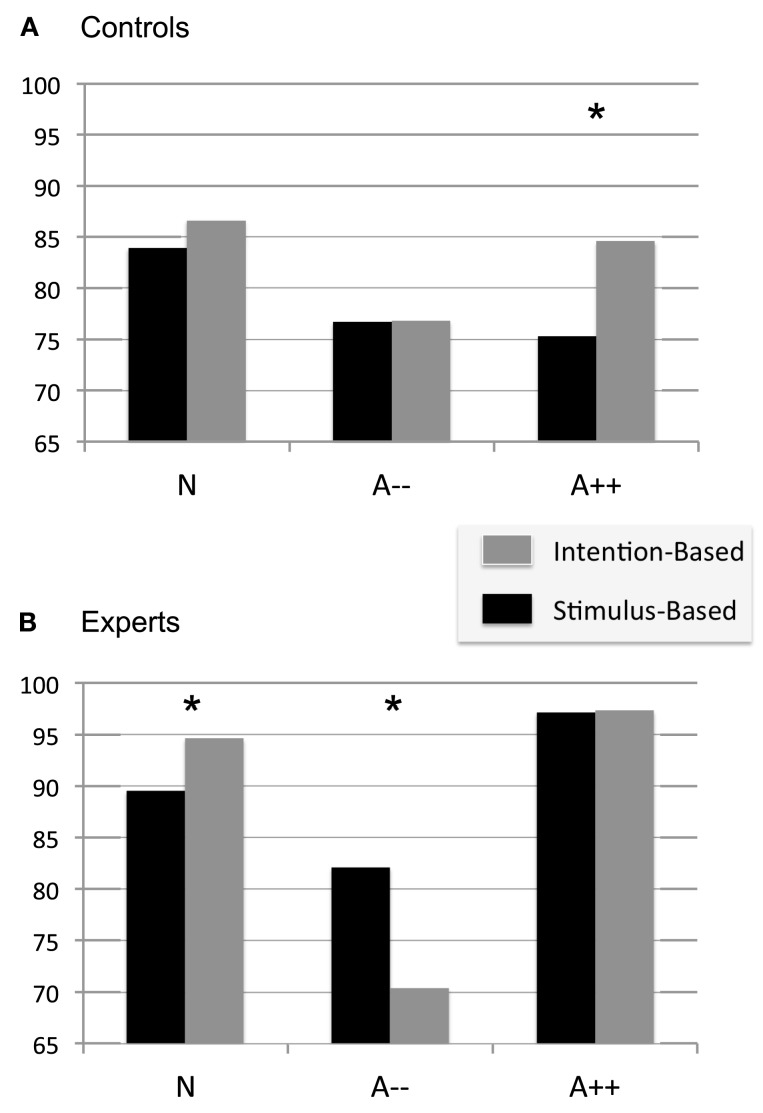
**Bar charts of the mean group correlations between the grip force used on the LIFT trials and the grip force used on the REPRODUCE-trials**. Results are presented in function of action mode and of experimental groups. **(A)** Adults with no experience in mindfulness meditation (Controls; *N* = 20); **(B)** Adults with daily experience in mindfulness practice (MBSR-experts; *N* = 10). Stars indicate a significant difference, at an alpha level set at 0.05.

In the second series of analyses, we considered the correlation between the GF levels used during the LIFT trial and the motor judgment that was given by the subjects immediately afterward, during the REPRODUCE trial. The closeness of fit was taken as an indicator of the level of motor consciousness reached by each participant and thus, correlation values were calculated at an individual level for each experimental condition. A repeated measures ANOVA was then used to reveal the effects of Mode (stimulus-based; intention-based) and Attention (Neutral; Diminished; Heightened) on the levels of motor consciousness in function of expertise in MBSR meditation techniques. Correlation values (*r*^2^) are reported in % of variance explained, and are presented in Figure 2A, for the controls and Figure 2B for the MBSR-experts.

Participants were able to reproduce movement kinetics with similar force modulation patterns in the LIFT and in the REPRODUCE trials, under all conditions. However, their level of motor consciousness varied significantly from one individual to another with correlation values ranging from *r*^2^ = 43.1% (lowest of the controls) to *r*^2^ = 98.9% (the highest of the MBSR-experts). Motor consciousness was overall greater in the MBSR-experts (*r*^2^ = 87.4 SD 2.2%) than in the controls (*r*^2^ = 80.1 SD 4.3%). Results are thus presented for each group separately in the following section.

For the controls, motor consciousness was similar for the two action modes [*F*(1, 38) = 2.302; *p* = 0.146], and the effect of attention failed to reach significance [*F*(1, 38) = 2.807; *p* = 0.073]. But if anything, motor consciousness was worse in both Heightened and Diminished attention conditions compared to that seen in the Neutral condition (see Figure 2A).

For the MBSR-experts, both the effects of Attention [*F*(2, 18) = 23.466; *p* = 0.001] and the interaction Mode × Attention were significant [*F*(2, 18) = 7.570; *p* = 0.004]. In the Neutral condition, motor consciousness was greater in the intention-based mode (94.6 SD 1.0%) than in the stimulus-based mode (89.5 SD 1.8%). With Heightened attention, motor consciousness in the stimulus-based mode increased and there was then an absence of differences between intention-based (97.3 SD 0.9%) and stimulus-based modes of action planning (97.1 SD 0.5%). This result suggests that, contrary to the controls, mindfulness-based meditation helped participants increase the level of motor consciousness even for those actions that were programmed in a more automatic fashion (see Figure 2B). Finally, the dual task paradigm led to a significant loss of motor consciousness in both types of action modes, but the loss was significantly more drastic for those actions performed in the intention-based mode (70.4 SD 4.4%) than those planned in the stimulus-based mode (82.1 SD 4.3%). Under this Diminished condition, motor consciousness was of similar degree in the controls and the MBSR-experts.

### Self-evaluation of motor consciousness

When asked to provide a self-evaluation of their capabilities to perform the REPRODUCE trials, all subjects thought that they had performed under the stimulus-based mode rather well (7/10 on a Likert-type rating scale; range 5–9). All subjects reported having great doubts for those trials performed under the intention-based mode (3/10 on a Likert-type rating scale; range 2–5). For the effects of attention, the self-evaluation reports followed a similar pattern of results in both experimental groups. Whatever the level in MBSR meditation technique, participants thought to have done worse in the Diminished condition and best in the Heightened condition. To note, is the fact that professional musicians (*N* = 12) were those who were overall the most accurate in the self-evaluation exercise.

These results suggest that whatever the level of MBSR-expertise, one can reach a certain degree of motor consciousness of movement kinetics without being self confident about the accuracy of the judgment. These findings suggest a pre-reflective nature to the mechanism and stress furthermore the importance of using non-verbal tasks to investigate levels of consciousness reached for motor-sensory body experiences.

## Discussion

Considering the pre-reflective nature of body experiences, reports in the literature have defended the idea that only limited aspects of motor acts can be consciously perceived. Nevertheless, when using appropriate methods, i.e., non-verbal, it was possible here to show that individuals have in fact access to a significant extent of the motor-sensory content of motor actions. Our main findings are that (1) with free allocation of attention (neutral condition), adult individuals can remarkably reproduce force levels that are automatically scaled to an object’s weight. (2) The level of motor consciousness is severely impaired when attention is withdrawn. (3) Increasing the focus of top-down attention (heightened condition) does not increase motor consciousness in controls; on the contrary, in the case of automatic motor adjustments (stimulus-based actions), increased top-down attention can hinder the emergence of body awareness. (4) Contrasting results are obtained for mindfulness experts with enhanced motor consciousness under Heightened focus of attention for motor adjustments that were both voluntarily (intention-based) and automatically scaled (stimulus-based). These later results suggest that MBSR meditation is not simply an increased state of attention but may also play on the threshold of conscious perception for bottom-up motor-sensory information. In the following sections, we detail these results by considering the role of attention, intention, and mindfulness meditation, respectively in the theoretical context of a *tripartite model of consciousness*.

### An implicit measure to evaluate motor consciousness

Motor consciousness is by definition an “inner subjective state” (Searle, 2000) and thus, “it is not directly accessible from a third person viewpoint.” Following this idea, we asked subjects to focus on the dynamical rather than on the observational aspect of their motor output. The basic assumption of such a reproduction paradigm was that the movement characteristics that can be reproduced are those of which we are aware of, at a pre-reflective level, and that the modulation of attention resources will help emerge to consciousness only those body motor-sensory experiences, which possess a constructed representational content.

With freely oriented attention (neutral condition), participants were able to reproduce the force levels used with correlations (*r*^2^) greater than 70% of total variance. This observation indicated that the reproduction task was based on a true content of body experience. It has been assumed in the literature that subjects’ perceptual awareness (their explicit knowledge of *action goal*) is equivalent to their motor awareness (their explicit knowledge of their *physical response* – for further discussion, see Coello and Delevoye-Turrell, 2007; Johnson et al., 2002). But, this is not the case as we demonstrated in the present study that all subjects had good reproduction capabilities without explicit knowledge of action outcome. This important result confirms a previous study that also used manual responses to assess motor consciousness more accurately (Johnson and Haggard, 2005).

### The importance of attention for motor consciousness

Our findings confirmed the hypothesis that attention resources are required to reach good levels of motor consciousness. In a dual task paradigm, subjects were required to perform a cognitive highly demanding count down task while preparing and performing the lift action. GF levels were reproduced with greater errors under this dual task condition (70–75%) compared to that observed under the single task condition (85–95%). These results suggest that attention allocation during action preparation and execution is required in order to access the content of body motor-sensory experiences, at a later moment. Without these cognitive resources, one loses in our simple case up to 25% of information content. This may explain why pathologies associated to attention deficits are often characterized by abnormal conscious experiences, e.g., schizophrenia (Davie and Freeman, 1961; Sass and Parnas, 2003; Voss et al., 2010) and bipolar patients (Bartolomeo, 2007; Lanyon and Denham, 2010).

Interestingly, and contrary to that hypothesized, subjects did not take advantage of the augmented attention allocation for better motor consciousness. If anything, augmented attention lead to a decrease in the accuracy of force reproduction. These results confirmed the conclusions reached in many sports oriented studies that declare that motor routines must be learned and performed without attention allocated to motor planning (Forkstam and Petersson, 2005; Janacsek and Nemeth, 2012). As such, it has been established that attention must be geared to external goals for high motor performance in order to maintain all attention away from those brain mechanism that automatically organize and execute action sequences (Wulf and Prinz, 2001; Wulf et al., 2010a). Hence, heightened attention for motor planning – through for example MBSR meditation techniques – may not be adequate for those actions that depend on brain processes that are by nature automatic and unconscious.

### Motor consciousness depends on the intentional state of action planning

It is the case that the negative effect of heightened attention on motor consciousness was observed in the controls only for those actions that required an automatic scaling of GF, on the basis of the true weight of the object, i.e., for stimulus-based planned actions (Figure 2A). Indeed, in those cases for which force scaling was set on the basis of an intentional and explicit motor goal, augmented attention to the task-preparation and execution did not interfere with the overall process; The content of body experiences was accessed in the following trial as well as that observed in the neutral condition. These results confirm that (1) the mechanisms for the planning and execution of motor actions are of a different nature depending on the intentional state of the subjects and that (2) attention to action will have a different effect on motor consciousness depending on the explicit level in which the performer is engaged.

Other studies have also reported that the degree of motor consciousness depends on the subjects’ intentional state (Castiello et al., 1991; Beilock et al., 2002, 2006). For example, using a movement reproduction paradigm in a double-step pointing task, Johnson et al. (2002) had subjects follow a target (*pointing*) or voluntarily move in the opposite direction (*anti-pointing*). After each initial trial, an indicator of the subjects’ awareness was obtained by asking subjects to reproduce the movement they thought they had previously executed. Results confirmed that subjects were able to make rapid corrections to an ongoing pointing movement, in response to a target shift. For anti-pointing trials, the corrections occurred later than the corrections toward the target in standard pointing. This pattern of results is consistent with the idea that two different mechanisms are involved: (1) a relatively slow neuronal circuit via the frontal cortices for intentional corrections, (2) a faster parietal connection for automatic corrections (Day and Lyon, 2000).

The interesting finding however was that subjects were able to perceive and reproduce the trajectories of the pointing corrections even in absence of a conscious perception of a target shift, indicating once more a significant different between perceptual and motor consciousness. In addition, there was a net difference in the quality of the content of motor awareness. Indeed, for standard pointing (automatic pointing), subjects systematically underestimated their correction-capabilities, with important time delays (>30 ms) and diminished awareness of the spatial characteristics of the correction. In contrast, for the anti-pointing trials (intentional pointing), subjects reproduced the corrections close to that truly performed with very little awareness time-delay. Thus, subjects had a better conscious recollection of those corrections made in the intention-based mode of action correction. Our findings lead to a similar conclusion but in the force domain of motor control, during a highly ecological task of manipulating an object with a precision grip. Hence, it is possible to suggest a generalization principle that subjects possess different levels of motor consciousness of their body in action, depending upon the nature of the planning-mode used to prepare and execute that action. More specifically, intention-based actions would be sub-conscious but would be more accessible to a conscious state of processing than the more automatically triggered movements.

### Mindfulness is not allocation of attention to the content of body experiences

An often-cited definition of mindfulness is *paying attention in a particular way*: on purpose, in the present moment and non-judgmentally (Kabat-Zinn, 1994). It has been proposed that this definition embodies three principle axioms (Shapiro et al., 2006): (1) *on purpose or intention*, (2) *paying attention or attention*, (3) *in a particular way or attitude*. As such, intention, attention, and attitude would be three interwoven aspects of a single cyclic process and would occur simultaneously. Mindfulness would be this moment-to-moment process. Very little work has been geared to the proposal of a comprehensive theoretical model of what happens under mindfulness meditation. Nevertheless, Hölzel and collaborators have recently proposed that mindfulness meditation would play upon several components and would especially change the cortical relation between (1) the anterior cingulated cortex for attention regulation (Hölzel et al., 2007) and (2) gray matter concentration in the temporo-parietal junction (Hölzel et al., 2011), which may modify the levels of body awareness.

Experimentally, meditation has shown to provide individuals with the capacity to increase cognitive resources both in healthy subjects (Jain et al., 2007; van den Hurk et al., 2010) and in pathological patients (Britton et al., 2010; Crane-Okada et al., 2012). Meditation has also revealed to increase body awareness (Kerr et al., 2008). In the present study, results are in line with these general ideas and showed that already in the neutral condition, participants with high experience in MBSR meditation techniques revealed greater capacities in reproducing a force level than those who had no meditation expertise.

Interestingly, even for MBSR-experts, when attention resources were reduced through dual task manipulation, subjects revealed weaker levels of motor consciousness than that measured in the neutral condition. This effect was drastic for the intention-based mode that impaired motor awareness to such an extent that controls and MBSR-experts revealed similar levels of motor consciousness under this condition only. Finally, in the heightened attention condition. Results revealed that the effect of *paying attention in a particular way* increased motor consciousness both in the intention-based mode and in the stimulus-based mode of action planning. This was possible to such an extent that performances reached almost perfection, with correlation values being all over 90%, and reaching 99% in 2 of the participants. It would now be important in forthcoming studies to further confirm the present results (1) by testing a larger group of meditation experts and (2) by controlling the type and level of meditation expertise developed on a daily basis.

In the final section, we discuss our results in the light of a possible neuro-cognitive model that may help gain a better understanding of the contrasting effects of attention and meditation on motor consciousness.

### A tripartite model to explain the effects of meditation on the emergence of subliminal sensory information

Instead of the classical binary separation between non-conscious and conscious processing, Dehaene et al. (2006) introduced a tripartite distinction between subliminal, preconscious, and conscious processing. More specifically, the key idea is that, within non-conscious states, it makes a major difference whether the stimuli invisibility is due to a limitation in bottom-up stimulus strength, or by the temporary withdrawal of top-down attention. The first case corresponds to subliminal processing; the second to preconscious processing. Following this idea, motor consciousness would be the resultant of the interaction between bottom-up mechanism for body sensations (depending on the strength of motor-sensory content) and the amount of top-down attention allocated to the task, at a given moment in time.

**Figure 3:**
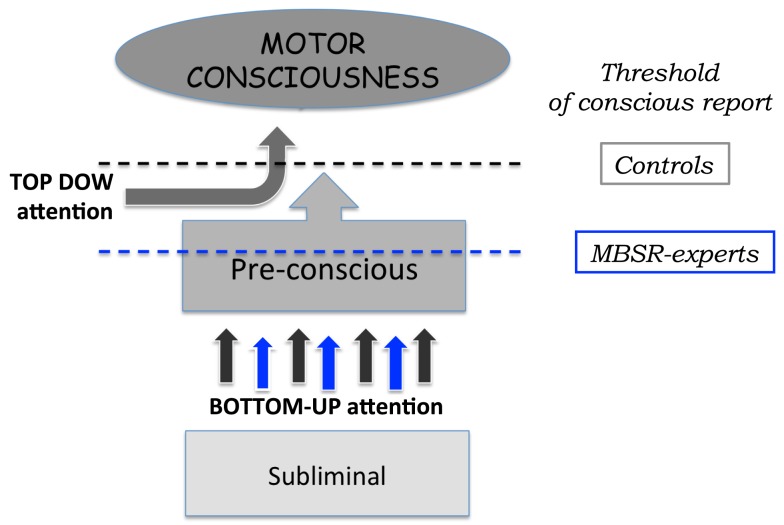
**Schematic illustration of the differentiated role of top-down and bottom-up attention for movement planning and execution**. Different levels of motor-sensory content are proposed in this tripartite model of motor consciousness. At the subliminal level, subjects have no access to the motor-sensory content; through allocation of bottom-up attention, information is transferred from the subliminal to the pre-conscious level of motor consciousness. The information available at this pre-conscious level can be transferred to the conscious level through the allocation of top-down resources. It is further proposed that MBSR expertise can enhance motor consciousness through two different mechanisms: increased synergies for more information transfer from subliminal to pre-conscious levels: better attention focus for lower threshold levels of conscious report.

It has been proposed that the subliminal level of processing (etymologically “below the threshold”) would be a condition for which information is inaccessible to consciousness because this bottom-up activation is insufficient to trigger a large-scale reverberating state, in a global network of neurons, with long-range axons. The preconscious level would be a neural process that potentially carries enough activation for conscious access, but is temporarily buffered in a non-conscious store because of a lack of top-down attentional amplification, e.g., owing to transient occupancy of the central workspace system during dual task conditions (Sigman and Dehaene, 2005, 2008). Even strong sensory stimuli could remain temporarily preconscious. With top-down attention focus, these preconscious stimuli could become conscious and thus, be explicitly reported by a subject. At the neurocomputational level, preconscious processing is proposed to involve resonant loops within medium range connections, which maintain the representation of a motor-sensory content temporarily active in a sensory buffer for a few hundred milliseconds. A preconscious stimulus might ultimately achieve conscious access once the central workspace is freed. It might however never gain access to conscious processing if the preconscious buffer is erased before receiving sufficient top-down attention. An illustration of this tripartite distinction is proposed in Figure 3.

We propose that the experimental data reported in the present study can be placed within this framework of consciousness. For actions performed in the stimulus-based mode, GF is automatically scaled to the object weight. Little bottom-up attention is geared to the task and thus, motor-sensory afferences from the gripping fingers arrive and remain primarily in the subliminal state. When during the REPRODUCE trial, subjects try to indicate the level of GF used in the previous LIFT trial, large reproducing errors are performed because little motor-sensory content has reached levels of explicit motor consciousness, even after focusing top-down attention to the task. This mechanism would explain why subjects know that they are agent of the action, and they know what final goal they achieved (*“I lifted the object to this height”*) but they have very little sensation content of the acting fingers upon the object. Because of the automaticity of the gripping action, with heightened top-down attention, large interferes occur between top-down and bottom-up attentional systems, which in turn affects the transition of motor-sensory information from subliminal to the preconscious state of processing.

For actions performed in the intention-based mode, top-down attention is used to scale voluntarily the GF levels in accordance to the internal representation of the weight of the imaginary object. In this case, in addition to bottom-up attention for muscle activation, top-down attention is also used to maintain a vivid representation to guide action planning and execution. This double attention activity would allow for a rich motor-sensory content to be buffered in the preconscious level of processing. With larger and more precise content, the REPRODUCE trial would be performed more accurately. As confirmed by our data, the heightened attention condition may not change performance outcome as top-down attention is allocated to the task whether explicitly through instruction or implicitly due to task demands. In the present study, the absence of differences in the controls between motor consciousness for intention and stimulus-based actions in the neutral condition may be due to the simplicity of the gripping task. Hence, our interpretations need now to be verified by replicating the present findings in a larger sample group and especially, using a more complex task that associates new experiences of whole body movements.

Finally, the power of meditation would lie within the possibility to increase both types of attention in order to optimize the quantity and quality of motor-sensory content. For the bottom-up circuit, meditation would lead to increased synergies during muscle activation, which in turn would code sensations directly into the preconscious level of information processing. As such, movements that are planned in the stimulus-based mode would be associated to sensory contents that are as vivid than that obtained for those actions planned intentionally. With more focused energies in the top-down areas of the brain, a lowering of the threshold level of conscious report could in addition occur in the most experts (Figure 3 – right), leading to a global level of motor consciousness close to perfection. Using a combination of attention paradigms, Jensen et al. (2012) have recently suggested a similar interpretation of MBSR-enhancement in reaction time based measures.

## Conclusion

The results reported here confirm that mindfulness increases the sensory experiences of body during motor action execution. The intentional state in which the action is produced plays an important role in the level of motor consciousness that subjects can achieve. This is probably due to the fact that intention-based actions require that plan-related representations be internally generated. With more top-down attention, greater amounts of sensory information are buffered at a preconscious level of motor-sensory processing. As described in the tripartite model of consciousness (Dehaene et al., 2006), top-down attention would play the role of an amplifier of bottom-up sensations that remain nevertheless preconscious in most everyday activities. Meditation techniques significantly enhance bottom-up sensory information processing of ongoing movements, enabling sensory information to transfer directly from subliminal to preconscious levels of the brain. When top-down attention is then directed toward these preconscious senses of body experiences, total consciousness of our body in action can emerge for even the simplest of movements.

## Conflict of Interest Statement

The authors declare that the research was conducted in the absence of any commercial or financial relationships that could be construed as a potential conflict of interest.
